# Association Between Benzodiazepine Use With or Without Opioid Use and All-Cause Mortality in the United States, 1999-2015

**DOI:** 10.1001/jamanetworkopen.2020.28557

**Published:** 2020-12-09

**Authors:** Kevin Y. Xu, Sarah M. Hartz, Jacob T. Borodovsky, Laura J. Bierut, Richard A. Grucza

**Affiliations:** 1 Health and Behavior Research Center, Department of Psychiatry, Washington University School of Medicine in St Louis, St Louis, Missouri; 2 Alvin J. Siteman Cancer Center, Barnes Jewish Hospital, Washington University School of Medicine in St Louis, St Louis, Missouri; 3 Center for Health Outcomes Research, St Louis University, St Louis, Missouri

## Abstract

**Importance:**

Although overall rates of opioid use have been plateauing, coprescriptions of benzodiazepines and opioids have increased greatly in recent years. It is unknown whether this combination is an independent risk factor for all-cause mortality as opposed to being more frequently used by persons with a baseline elevated risk of death.

**Objective:**

To evaluate whether benzodiazepine use, with or without opioid use, is associated with increased all-cause mortality relative to the use of low-risk antidepressants.

**Design, Setting, and Participants:**

This retrospective cohort study used a large, nationally representative US data set (the National Health and Nutrition Examination Surveys [NHANES]) from 1999 to 2015. Eight cycles of NHANES data were used, spanning 37 610 person-years of follow-up time among 5212 individuals. Statistical analysis was performed from August 24, 2019, through May 23, 2020.

**Exposures:**

The primary exposure variable was benzodiazepine and opioid coprescriptions. Individuals taking selective serotonin reuptake inhibitors (SSRIs) served as an active comparator reference group.

**Main Outcomes and Measures:**

All-cause mortality was obtained via linkage of NHANES to the National Death Index. Propensity scores were calculated from covariates associated with sociodemographic factors, comorbidities, and medication use for more than 1000 prescription types. Propensity score–weighted mortality hazards were calculated from Cox proportional hazards regression models.

**Results:**

Of 5212 participants aged 20 years or older (1993 men [38.2%]; mean [SD] age, 54.8 [16.9] years) followed up for a median of 6.7 years (range, 0.2-16.8 years), 101 deaths (33.0 per 1000 person-years) occurred among those receiving cotreatment, 236 deaths (26.5 per 1000 person-years) occurred among those receiving only benzodiazepines, and 227 deaths (20.2 per 1000 person-years) occurred among SSRI recipients taking neither opioids nor benzodiazepines. After propensity score weighting, a significant increase in all-cause mortality was associated with benzodiazepine and opioid cotreatment (hazard ratio, 2.04 [95% CI, 1.65-2.52]) and benzodiazepines without opioids (hazard ratio, 1.60 [95% CI, 1.33-1.92]). Subgroup analyses revealed an increased risk of mortality for individuals receiving cotreatment who were 65 years or younger but not for those older than 65 years; similar findings were observed for those receiving benzodiazepines without opioids.

**Conclusions and Relevance:**

This study found a significant increase in all-cause mortality associated with benzodiazepine use with or without opioid use in comparison with SSRI use. Benzodiazepine and opioid cotreatment, in particular, was associated with a 2-fold increase in all-cause mortality even after taking into account medical comorbidities and polypharmacy burden.

## Key Points

**Question:**

Is benzodiazepine use, with or without opioid use, an independent risk factor for all-cause mortality or a marker for underlying conditions associated with death?

**Findings:**

In this cohort study of 5212 individuals from a large, nationally representative data set, it was found that benzodiazepine use, with or without opioid use, was associated with a doubling in all-cause mortality risk in comparison with the use of low-risk antidepressants. These findings persisted even after adjustment for sociodemographic variables and comorbidity burden.

**Meaning:**

This study suggests that benzodiazepine and opioid cotreatment may confer an increased long-term mortality risk; targeted interventions are needed to decrease overprescribing.

## Teaser

This cohort study uses data from the National Health and Nutrition Examination Surveys to evaluate whether benzodiazepine use, with or without opioid use, is associated with increased all-cause mortality relative to the use of low-risk antidepressants.

## Introduction

Benzodiazepines are among the most widely used medications worldwide and are prescribed for common problems such as anxiety and insomnia.^1,2,3^ Up to 13% of US adults have reported benzodiazepine use in the last year,^4^ with ambulatory benzodiazepine prescriptions more than doubling in the last decade.^5^

Increasing benzodiazepine prescriptions have clinical relevance amid declining life expectancies and a global epidemic of opioid overdoses. When benzodiazepines are taken alone, long-term use is associated with falls, cognitive impairment, and life-threatening withdrawal.^1,3^ When taken together with opioids, benzodiazepines can further suppress breathing, a common cause of death from opioid overdose.^1,6,7^ The danger of benzodiazepine-opioid cotreatment is thus a timely issue, as 20% to 30% of all benzodiazepine recipients in the US are estimated to have an opioid coprescription^8^ and more than 30% of opioid overdose deaths are found to involve benzodiazepines.^9,10,11^ Despite a recent plateau in overall opioid use in the US,^8,12,13^ benzodiazepine-opioid coprescriptions continue to increase.^2,8,14,15,16^ It is tempting to infer elevated mortality risk attributable to benzodiazepines alone or in combination with opioids, but this topic has proven challenging to study. To date, it is not well understood if benzodiazepine use—whether with or without opioids—is associated directly with mortality, as opposed to serving as a marker for unmeasured underlying conditions that are the true factors associated with excess deaths.

In the case of benzodiazepine use without opioids, data on its long-term mortality risks are marked by inconsistent findings. Although some studies have found substantial mortality associated with benzodiazepine use without opioids,^17^ such that the hazards of taking benzodiazepines more than 30 times per month were akin to smoking 1 to 2 packs of cigarettes per day,^18,19^ other studies have not supported these findings,^20^ with several analyses suggesting that benzodiazepine-associated mortality may vary based on age or follow-up time.^17,20^ Furthermore, these studies have often been limited to employed, middle-aged populations in commercial insurance databases,^20^ which may exclude individuals with a heavier comorbidity burden who initiate benzodiazepines.^20,21^

In the case of benzodiazepine-opioid cotreatment, the literature base is similarly limited. Although recent data have depicted an increased risk of emergency department visits^16^ and opioid overdose deaths in patients with such coprescriptions,^22,23^ these findings have often been limited to patients surviving opioid overdoses,^16^ subpopulations such as veterans,^24^ and short-term follow-up.^16,24^

Our study addresses the existing research gap by evaluating the association of benzodiazepine use with or without opioid coprescriptions with long-term all-cause mortality using a large nationally representative data set in the US linked to the National Death Index (NDI), spanning approximately 15 years of follow-up time (1999-2015).

## Methods

We used the publicly available National Health and Nutrition Examination Surveys (NHANES) to obtain a nationally representative sample of approximately 5000 US participants per 2-year cycle. The NHANES data were collected from 1999 until 2014, with mortality data extending through 2015. Face-to-face interviews and physical examinations were conducted in participants’ homes by interviewers, physicians, and medical technicians (net response rate, 75%), in addition to physical examinations conducted in mobile examination centers (net response rate, 70%). Full details on NHANES are described elsewhere.^8,25^ As all data were completely deidentified, our analyses did not require human participants review by the Washington University institutional review board. Consent was waived because all data were deidentified. The Strengthening the Reporting of Observational Studies in Epidemiology (STROBE) reporting guideline was followed.

### Participants

**Figure 1.**
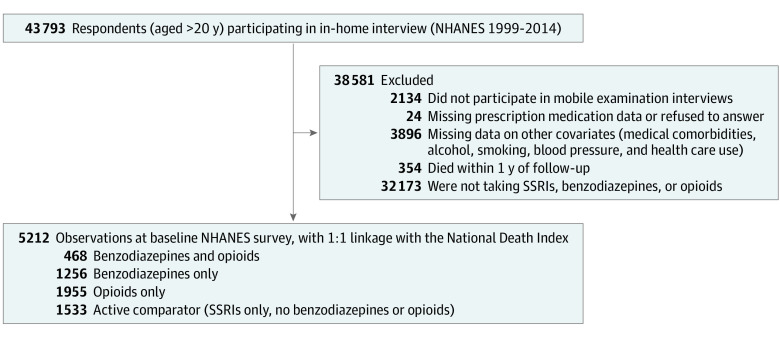
Study Inclusion and Exclusion Flowchart Derivation of the study cohort during follow-up, with 1:1 linkage to the National Death Index. We excluded those who did not participate in mobile examination interviews, were missing medication data or refused to answer, or were missing data on other covariates included in our propensity scores. We also excluded those who died within 1 year of follow-up or were not taking selective serotonin reuptake inhibitors (SSRIs), benzodiazepines, or opioids. NHANES indicates National Health and Nutrition Examination Surveys.

Eligible participants were aged 20 years or older. The observation window during which death was ascertained began immediately after individuals were surveyed in the in-home interview. Out of 43 793 respondents who participated in the in-home interview and physical examinations, we excluded 354 persons who died within 1 year after recruitment to reduce bias arising from benzodiazepine prescriptions for participants with terminal illness.^17^ We further excluded individuals who lacked data on any prescription medications (n = 24), mobile examinations (n = 2134), and study covariates (n = 3896) (Figure 1). We also excluded those without prescriptions for selective serotonin reuptake inhibitors (SSRIs), benzodiazepines, or narcotics (n = 32 173). Our final analytic sample of 5212 individuals was linked to the NDI, which contains data on date and underlying cause for more than 99% of all US deaths,^26,27,28^ with a sensitivity and specificity both estimated to be greater than 97%.^29^ Linked mortality data extend from January 1, 1999, until time of death or censorship at December 31, 2015, whichever occurred first. If no match was found with the NDI, participants were assumed to be alive as of that date.

### Ascertainment of Medication Use Data

Data were collected by NHANES on prescription medications taken, spanning both short-term and long-term prescriptions. Participants were required to show examiners the containers of all medications used in the last month. The percentage of respondents who provided physical evidence of medication use ranged from 77.7% (11 512 of 14 817) in the 2009-2010 cycle to 94.5% (13 252 of 14 019) in the 2013-2014 cycle, with medication use ascertained via self-report for the remaining individuals who could not produce prescription bottles. Of 43 793 respondents participating in in-home interviews, fewer than 0.05% (n = 24) were missing prescription medication data or refused to answer.^12^ Medication data were limited to prescriptions for 30 days or more to exclude short-term medications for acute medical issues.^12^

Medication use was matched to Lexicon Plus by NHANES, which used Cerner Multum Inc to classify medications by therapeutic medication categories.^8,12^ We tabulated 23 total benzodiazepines, encompassing short-acting agents, long-acting medications, and selective benzodiazepine receptor modulators (“Z-drugs” [eg, zolpidem]). Based on established methods in previous studies,^8,12^ opioid narcotics included more than 60 generic and brand-name prescription pain medications^12^ (full classification scheme details in eTable 1 in the Supplement). Because selective benzodiazepine receptor modulators have been found to share similar adverse effects as benzodiazepines,^30^ benzodiazepines and benzodiazepine receptor modulators were grouped together.

### Covariates and Confounders

To account for confounding medical and psychiatric comorbidities that may be associated with increased mortality in the population of individuals receiving benzodiazepines, we developed propensity score weights based on the estimated probability of benzodiazepine prescription status, using covariates found to be prevalent among persons receiving benzodiazepines^3^; these covariates spanned sociodemographic factors, health care use variables, psychiatric comorbidities, and medication use data for more than 1000 unique prescriptions. Full details are described in the eMethods in the Supplement.

To increase overlap of measured characteristics between benzodiazepine-opioid users and nonusers, we used an active comparator group of participants who were not taking benzodiazepines or opioids and had an active prescription for SSRIs. This is a validated approach taken to address unobserved confounding factors given SSRIs’ overlapping indications with benzodiazepines without any known association with all-cause mortality in adults.^20,31^ We selected SSRIs as our active comparator rather than non-SSRI antidepressants owing to substantial heterogeneity in non-SSRIs’ indications for use in comparison with benzodiazepines (ie, tricyclic antidepressants for headache and serotonin norepinephrine reuptake inhibitors for neuropathic pain)^20^ as well as increased morbidity and mortality associated with monoamine oxidase inhibitors and tricyclic antidepressants.^32^

### Statistical Analysis and Sensitivity Analyses

Statistical analysis was performed from August 24, 2019, through May 23, 2020. We calculated the overall rate of all-cause mortality per 1000 person-years, stratified by follow-up time (<50th percentile vs ≥50th percentile of follow-up time) and age (20-65 vs ≥65 years). We used the Cox proportional hazards regression model to estimate crude mortality hazards in comparing the active comparator group with exposure groups 1 and 2. Adjusted mortality hazards were then obtained after applying propensity score weighting, with SE estimates calculated via bootstrapping with 1000 replications (with a single imputation used within each sampling) to adjust for additional sources of variability introduced by the propensity score weighting process as well as multiple imputation. As previously described,^8^ NHANES reporting guidelines consider estimates to be statistically reliable with a sample size of more than 420.

To further evaluate robustness of our findings, we conducted stratified analyses by follow-up time (<50th percentile vs ≥50th percentile of follow-up time) and age (20-65 vs ≥65 years). Because NHANES does not provide data on temporality of health conditions and medications, we developed an alternative propensity score with more than 650 non–central nervous system (CNS) medications excluded (ie, cardiovascular, metabolic, and anti-infective medications) and repeated all analyses after limiting medications in our propensity scores to only those used to treat chronic CNS disorders. All analyses were performed using SAS, version 4.2 (SAS Institute Inc). All *P* values were from 2-sided tests and results were deemed statistically significant at *P* < .05.

## Results

### Sample Characteristics

Our sample consisted of 5212 participants (1993 men [38.2%]; mean [SD] age, 54.8 [16.9] years; 3308 White [63.5%]), with baseline characteristics depicted in Table 1. Of all participants, 468 (9.0%) were prescribed both opioids and benzodiazepines, 1256 (24.1%) were prescribed benzodiazepines only, and 1955 (37.5%) were prescribed opioids only. A total of 1533 individuals (29.4%) had a prescription for neither opioids nor benzodiazepines but were taking SSRIs. In comparison with the SSRI group, those receiving benzodiazepine-opioid coprescriptions tended to be older (mean [SD] age, 56.1 [14.0] vs 53.8 [17.1] years), be male (171 [36.5%] vs 483 [31.5%]), and have a lower income (poverty to income ratio >2, 159 [34.0%] vs 837 [54.6%]), with a higher prevalence of smoking (183 [39.1%] vs 341 [22.2%]), disability (311 [66.5%] vs 447 [29.2%]), worsening health (155 [33.1%] vs 229 [14.9%]), hospitalization in the last year (159 [34.0%] vs 278 [18.1%]), and prescription medication use across both CNS and non-CNS medications, paralleling a higher prevalence of these characteristics in absolute standardized differences seen in eTable 2 in the Supplement. We used an absolute standardized difference of less than 0.1 to denote negligible difference in covariate balance between groups^20,33^ (eTable 2A, 2B, 2C, 2D, and 2E in the Supplement).

**Table 1.** Baseline Characteristics of Study Participants^a^

Characteristic	No. (%)					*P* value		
	All participants (N = 5212)	BZDs plus opioids (n = 468 [9.0%])	BZDs only (n = 1256 [24.1%])	Opioids only (n = 1955 [37.5%])	Neither (SSRIs) (n = 1533 [29.4%])	BZDs plus opioids vs neither (SSRIs)	BZDs vs neither (SSRIs)	Opioids vs neither (SSRIs)
Age, mean (SD), y	54.8 (16.9)	56.1 (14.0)	57.9 (16.7)	53.5 (17.1)	53.8 (17.1)	.02	<.001	.18
Aged 60-70 y	1058 (20.3)	103 (22.0)	241 (19.2)	421 (21.5)	293 (19.1)	.17	.96	.08
Male	1993 (38.2)	171 (36.5)	463 (36.9)	876 (44.8)	483 (31.5)	.04	.003	<.001
College graduate	900 (17.3)	49 (10.5)	235 (18.7)	262 (13.4)	354 (23.1)	<.001	.005	<.001
Poverty to income ratio								
1-2	1353 (26.0)	140 (29.9)	329 (26.2)	530 (27.1)	354 (23.1)	.005	.06	.008
>2	2402 (46.1)	159 (34.0)	616 (49.0)	790 (40.4)	837 (54.6)	<.001	.002	<.001
White	3308 (63.5)	322 (68.8)	859 (68.4)	1055 (54.0)	1072 (69.9)	.64	.38	<.001
Partnered	2990 (57.4)	251 (53.6)	702 (55.9)	1131 (57.9)	906 (59.1)	.04	.08	.59
Smoking	1427 (27.4)	183 (39.1)	292 (23.2)	611 (31.3)	341 (22.2)	<.001	.52	<.001
Hypertension	2228 (42.7)	244 (52.1)	545 (43.4)	797 (40.8)	642 (41.9)	<.001	.42	.53
Hyperlipidemia	1502 (28.8)	141 (30.1)	395 (31.4)	502 (25.7)	464 (30.3)	.51	.98	.06
Stroke	406 (7.8)	54 (11.5)	95 (7.6)	131 (6.7)	126 (8.2)	.03	.53	.09
Myocardial infarction	429 (8.2)	50 (10.7)	112 (8.9)	149 (7.6)	118 (7.7)	.04	.24	.93
Diabetes	953 (18.3)	93 (19.9)	205 (16.3)	391 (20.0)	264 (17.2)	.19	.47	.04
Congestive heart failure	356 (6.8)	44 (9.4)	89 (7.1)	127 (6.5)	96 (6.3)	.02	.38	.78
Pulmonary disease	1486 (28.5)	183 (39.1)	330 (26.3)	551 (28.2)	422 (27.5)	<.001	.54	.65
Liver disease	353 (6.8)	47 (10.0)	80 (6.4)	139 (7.1)	87 (5.7)	<.001	.44	.09
Arthritis	2665 (51.1)	337 (72.0)	567 (45.1)	1126 (57.6)	635 (41.4)	<.001	.06	<.001
Kidney disease	286 (5.1)	36 (7.7)	68 (5.4)	122 (6.2)	60 (3.9)	<.001	.06	.002
Cancer	779 (14.9)	99 (21.2)	221 (17.6)	276 (14.1)	183 (12.0)	<.001	<.001	.06
Regular drinking	1290 (24.8)	83 (17.7)	330 (26.3)	446 (22.8)	431 (28.1)	<.001	.28	<.001
Antimicrobial drugs	582 (11.2)	71 (15.2)	102 (8.1)	294 (15.0)	110 (7.2)	<.001	.19	<.001
Hormonal agents	1218 (23.4)	129 (27.6)	337 (26.8)	357 (18.3)	395 (25.8)	.44	.53	<.001
Anticonvulsants	580 (11.1)	104 (22.2)	127 (10.1)	233 (11.9)	116 (7.6)	<.001	.02	<.001
Any analgesics	1124 (21.6)	126 (26.9)	236 (18.8)	509 (26.0)	253 (16.5)	<.001	.11	<.001
Muscle relaxants	532 (10.2)	114 (24.4)	68 (5.4)	299 (15.3)	51 (3.3)	<.001	.007	<.001
Gastrointestinal agents	1512 (29.0)	201 (43.0)	381 (30.3)	534 (27.3)	396 (25.8)	<.001	.008	.33
Cardiac or metabolic medications	2983 (57.2)	315 (67.3)	782 (62.3)	1025 (52.4)	861 (56.2)	<.001	.001	.03
Respiratory medications or antihistamines	1056 (20.3)	154 (32.9)	234 (18.6)	395 (20.2)	273 (17.8)	<.001	.57	.07
>2 CNS medications	1070 (20.5)	186 (39.7)	250 (19.9)	435 (22.3)	199 (13.0)	<.001	<.001	<.001
<5 Non-CNS medications	993 (19.1)	97 (20.7)	263 (20.9)	333 (17.0)	300 (19.6)	.58	.37	.05
Antidepressants (SNRIs, MAOIs, or TCAs)	396 (7.6)	84 (18.0)	130 (10.4)	142 (7.3)	40 (2.6)	<.001	<.001	<.001
Other antidepressants	510 (9.8)	69 (14.7)	153 (12.2)	131 (6.7)	157 (10.2)	.007	.10	<.001
Antipsychotics	280 (5.4)	34 (7.3)	102 (8.1)	39 (2.0)	105 (6.9)	.76	.20	<.001
Any hospitalization in <1 y	1267 (24.3)	159 (34.0)	299 (23.8)	531 (27.2)	278 (18.1)	<.001	<.001	<.001
Any psychiatric visit in <1 y	1125 (21.6)	129 (27.6)	351 (27.9)	217 (11.1)	428 (27.9)	.88	.97	<.001
Good current health	3073 (59.0)	181 (38.7)	785 (62.5)	1045 (53.5)	1062 (69.3)	<.001	<.001	<.001
Require special health equipment	1154 (22.1)	173 (37.0)	202 (16.1)	540 (27.6)	239 (15.6)	<.001	.72	<.001
Disabled	2096 (40.2)	311 (66.5)	453 (36.1)	885 (45.3)	447 (29.2)	<.001	<.001	<.001
Regular ED care	113 (2.2)	11 (2.4)	17 (1.4)	69 (3.5)	16 (1.0)	.04	.46	<.001
Worsening health	1148 (22.0)	155 (33.1)	269 (21.4)	495 (25.3)	229 (14.9)	<.001	<.001	<.001
>2 Overnight hospitalizations in <1 y	444 (8.5)	64 (13.7)	100 (8.0)	181 (9.3)	99 (6.5)	<.001	.12	.003

Abbreviations: BZD, benzodiazepine; CNS, central nervous system; ED, emergency department; MAOI, monoamine oxidase inhibitor; SNRI, serotonin norepinephrine reuptake inhibitor; SSRI, selective serotonin reuptake inhibitor; TCA, tricyclic antidepressant.

^a^ Two-sided *P* values show results of univariate comparisons between participants taking neither BZDs nor opioids (active comparator: SSRIs) and those taking BZDs plus opioids, BZDs only, and opioids only. Categorical variables were tested with the χ^2^ test. Continuous variables were tested with the Wilcoxon rank sum test.

### Survival Analysis

The median time to death or censoring was 6.7 years (range, 0.2-16.8 years) for all participants (eTable 3 in the Supplement). A total of 1423 of 5212 participants (27.3%) had follow-up times exceeding 10 years, with 37 610 person-years of follow-up time among all participants. Of 892 deaths that occurred in total (23.7 events per 1000 person-years), 101 (33.0 per 1000 person-years) occurred among those receiving cotreatment, 236 (26.5 per 1000 person-years) occurred among those using only benzodiazepines, 328 (22.8 per 1000 person-years) occurred among those using only opioids, and 227 (20.2 per 1000 person-years) occurred in the active comparator group of SSRI recipients taking neither benzodiazepines nor opioids (eTable 3 in the Supplement).

**Figure 2.**
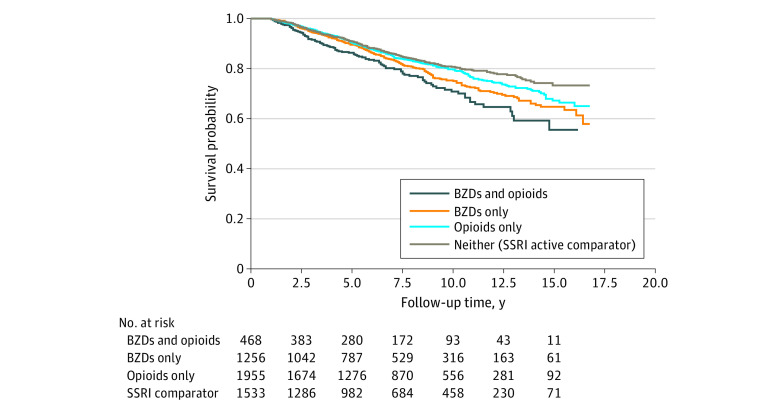
Unadjusted Kaplan-Meier Curves for Survival Probability, With Follow-up in Years Kaplan-Meier curves of survival probability stratified by benzodiazepines (BZDs) plus opioids, BZDs only, opioids only, and neither BZDs nor opioids (active comparator, selective serotonin reuptake inhibitors [SSRIs]). We excluded all participants who died within 1 year of follow-up.

Figure 2 illustrates unadjusted Kaplan-Meier curves for survival probability at the start of follow-up, for which unadjusted log-rank tests showed that all-cause mortality was significantly elevated in the benzodiazepine-opioid cotreatment group in comparison with those taking benzodiazepines only, opioids only, and SSRI recipients taking neither (χ^2^ = 22.41; *P* < .001). Among those who were 65 years or younger, we calculated an event rate of 10.3 per 1000 person-years in comparison with 59.9 per 1000 person-years among their peers older than 65 years (eTable 3 in the Supplement).

In unadjusted Cox proportional hazards regression models, patients receiving benzodiazepines, both with and without opioids, exhibited an elevated risk of death compared with SSRI recipients receiving neither opioids nor benzodiazepines (cotreatment group: hazard ratio [HR], 1.71 [95% CI, 1.34-2.19]; benzodiazepines without opioids group: HR, 1.36 [95% CI, 1.13-1.64]) (Table 2). After propensity score weighting, hazards of death remained elevated in those receiving cotreatment (HR, 2.04 [95% CI, 1.65-2.52]). A smaller but nonetheless significant increase in all-cause mortality was associated with benzodiazepine use without opioids in adjusted analyses (HR, 1.60 [95% CI, 1.33-1.92]).

**Table 2.** Risk of All-Cause Mortality Associated With BZD With or Without Opioids in Unadjusted and Propensity Score–Weighted Analyses

Characteristic	Unweighted HR (95% CI)	*P* value	Weighted HR (95% CI)	*P* value
**BZDs only vs neither (active comparator, SSRIs)**				
All participants	1.36 (1.13-1.64)	.001	1.60 (1.33-1.92)	<.001
Age, y				
20-65	1.52 (1.06-2.18)	.02	1.81 (1.29-2.54)	<.001
>65	0.86 (0.68-1.07)	.17	0.84 (0.67-1.05)	.12
Follow-up time				
<50th percentile (6.6 y)	1.03 (0.82-1.31)	.79	1.17 (0.92-1.50)	.21
≥50th percentile (6.6 y)	1.81 (1.31-2.50)	<.001	2.17 (1.59-2.98)	<.001
**BZDs plus opioids vs neither (active comparator, SSRIs)**				
All participants	1.71 (1.34-2.19)	<.001	2.04 (1.65-2.52)	<.001
Age, y				
20-65	2.66 (1.77-4.00)	<.001	3.27 (2.40-4.47)	<.001
>65	1.10 (0.80-1.51)	.55	1.21 (0.86-1.70)	.28
Follow-up time				
<50th percentile (6.6 y)	1.21 (0.90-1.63)	.20	1.35 (1.04-1.76)	.02
≥50th percentile (6.6 y)	1.73 (1.09-2.75)	.02	1.93 (1.29-2.88)	.002

Abbreviations: BZD, benzodiazepine; HR, hazard ratio; SSRI, selective serotonin reuptake inhibitor.

### Subgroup and Sensitivity Analyses

We observed an increased risk of mortality associated with benzodiazepine-opioid coprescriptions among younger but not older participants (adjusted HR, 3.27 [95% CI, 2.40-4.47] vs 1.21 [95% CI, 0.86-1.70]), with similar findings among those receiving benzodiazepines without opioids (adjusted HR, 1.81 [95% CI, 1.29-2.54] vs 0.84 [95% CI, 0.67-1.05]) (Table 2). Among those older than 65 years in age-stratified analyses, we consistently observed no increased risk of death associated with cotreatment across all adjusted comparisons. Analyses were also repeated across participants of different follow-up periods, using the 50th percentile as a threshold between short-term and long-term follow-up strata. Among those receiving benzodiazepines without opioids, weighted analyses showed an increased risk of death in those with longer (≥50th percentile) but not shorter follow-up times (HR, 2.17 [95% CI, 1.59-2.98] vs 1.17 [95% CI, 0.92-1.50]). Weighted analyses showed similarly elevated hazards of death among those receiving benzodiazepine and opioid cotreatment regardless of follow-up time (<50th percentile: HR, 1.35 [95% CI, 1.04-1.76] vs ≥50th percentile: HR, 1.93 [95% CI, 1.29-2.88]).

In further sensitivity analyses, we calculated propensity scores that excluded non-CNS agents, with largely similar effect sizes across all comparisons (eTable 4 in the Supplement). In addition, we conducted a sensitivity analysis that included participants who survived fewer than 12 months after recruitment, with similar findings as in our analyses that excluded those who died within 1 year (eTable 5 in the Supplement).

## Discussion

This study illustrates that benzodiazepines, both by themselves and in combination with opioids, significantly increase the risk of all-cause mortality in comparison with SSRIs alone. In particular, the benzodiazepine-opioid coprescription group continued to show a nearly 2-fold increase in all-cause mortality even after taking into account medical comorbidities and polypharmacy burden in adjusted analyses.

Although benzodiazepines without opioids were associated with an overall increase in all-cause mortality in weighted analyses, these hazards of death were negligible among those with shorter as opposed to longer amounts of follow-up time. This finding may be consistent with existing literature^20^ suggesting that benzodiazepine use without opioids is more of a proxy for elevated short-term mortality risk than a factor directly associated with death. Furthermore, recent findings—focusing largely on acute as opposed to chronic benzodiazepine and opioid exposures—have suggested that fatalities associated with pure benzodiazepine overdose are rare and that the combination of sedative-hypnotic agents with opioids may be an important and understudied factor associated with mortality.^34^

We also conducted age-stratified analysis to clarify the burden of psychotropic medications in the elderly, particularly since earlier studies have found that older adults were more likely to receive benzodiazepines and opioids.^2,35,36^ Most studies that have identified a positive association between benzodiazepines and all-cause mortality were conducted in predominantly adult or young adult populations,^16,17,20^ which makes the mortality risk associated with benzodiazepine use in older adults a poorly understood area. We found no evidence of increased all-cause mortality stemming from benzodiazepine-opioid cotreatment in participants aged 65 years or older, which parallels similar null findings among elderly patients receiving benzodiazepines without opioids.^37^ There was a consistently elevated mortality risk in participants younger than 65 years, which may be associated with surging drug poisoning–related mortality that disproportionately affects younger US populations.^38^

### Strengths and Limitations

Our study has some strengths, including its use of an active comparator, which is associated with partial control for unobserved confounding. We further adjusted for observed confounding using propensity score weighting. Taken together, these methods adjust for many health behaviors and comorbidities that may be associated with increased benzodiazepine-associated mortality. This is particularly useful given concern that benzodiazepine-opioid cotreatment and all-cause mortality may share common antecedents or outcomes, potentially associated with confounding by indication and collider bias.^17^ Another study strength is the generalizability of NHANES data, which contain demographic groups that are frequently not included in Veterans Affairs data, as well as older individuals with a higher burden of medical and psychiatric comorbidities not included in insurance claims data.^16,20^ Finally, our analysis spans a longer follow-up period, with a median of nearly 7 years, than similar analyses,^16,20^ which may explain the elevated death rates observed in our study (26.5 per 1000 person-years) for benzodiazepines without opioids in comparison with the mortality rates found by Patorno et al^37^ (12 per 1000 person-years), whose mean follow-up period was 145 to 160 days.

There are also several limitations that need to be considered. First, despite the use of an active comparator and good propensity score balance achieved, residual confounding cannot be completely excluded. Furthermore, propensity score weighting does not ensure avoidance of collider stratification bias. Second, NHANES does not provide data differentiating on indication, duration, and dosage of benzodiazepine use. Although our study cannot differentiate between individuals newly initiating benzodiazepines and longer-term users, previous work has not found significant differences in hazards even after adjusting for higher dosage and prolonged exposure^20^ or duration of use.^16^ Nonetheless, because each participant is only assessed once, it is not possible to assess changing patterns of benzodiazepine use over time.

Third, we are unable to deduce if medication use and medical comorbidities in NHANES precisely preceded exposure to benzodiazepines, which is generally an assumption of propensity score approaches.^39^ Despite our inability to infer such temporal precedence, we contend that NHANES medication data serve as a good proxy for unmeasured comorbidities that likely preceded benzodiazepine exposure. To assess the possibility of overcorrection via inclusion of mediating variables, we developed an alternative propensity score that excluded more than 650 non–CNS-acting medications that yielded largely similar results to those from our main models.

Fourth, while we were careful to include nearly every psychiatric prescription medication within our propensity score along with measures of mental health resource use, NHANES does not provide data on whether participants met criteria for *DSM*-validated anxiety and depressive disorders, which are often treated with benzodiazepines and have been found to correlate with increased suicide risk. Countering this information, use of an active comparator reduces between-group differences in depression and anxiety that could not be addressed using measured covariates.

Fifth, NDI-linked NHANES data are not yet available for more recent years, limiting the study’s generalizability. As the opioid epidemic has evolved since 2015, the most current trends warrant further investigation. Sixth, the public-use NDI does not provide data on overdose or suicide-specific mortality. Despite this limitation, our focus on all-cause mortality has the advantage of avoiding misclassification and missing data that can occur in cause-specific mortality analyses. Furthermore, recent data^24^ suggest that nearly 90% of benzodiazepine-opioid cotreatment deaths were due to nonoverdose causes. Future studies will need to differentiate between benzodiazepine use and misuse while taking into account illicit drug use, which occurs frequently in patients using benzodiazepines, although preliminary data suggest that only 1.5% of patients with benzodiazepine prescriptions develop a substance use disorder.^21^

## Conclusions

Our findings suggest that benzodiazepine-opioid cotreatment and use of benzodiazepines without opioids are associated with long-term all-cause mortality in comparison with individuals who used low-risk antidepressants. We also found that benzodiazepine use (with or without opioids) was associated with increased mortality risk only among those 65 years or younger. Amid very few opioid prescribing guidelines targeting patients receiving sedative-hypnotics, benzodiazepine-opioid cotreatment poses a therapeutic dilemma for physicians. Concerted efforts by physicians, scientists, and policymakers are warranted to decrease overprescribing of these medications, identify patients at elevated risk, and ultimately implement targeted interventions.

## Notes

Supplement. **eMethods.** Overview of Propensity Score Derivation **eTable 1.** Classification Scheme of Benzodiazepines and Opioids **eTable 2.** Propensity Score Diagnostics for All Participants, as Well as Stratified by Age and Follow-up Time **eTable 3.** Follow-up Time and Event Rates Among Study Participants **eTable 4.** Sensitivity Analyses Depicting Risk of All-Cause Mortality Associated With Benzodiazepine/Opioid Co-Treatment and Benzodiazepine Monotherapy, With Removal of Non–Central Nervous System Medications **eTable 5.** Sensitivity Analyses Depicting Risk of All-Cause Mortality Associated With Benzodiazepine/Opioid Co-Treatment and Benzodiazepine Monotherapy, With Inclusion of Participants Who Died Within 1 Year
