# Supplementary material for: Association Between Benzodiazepine Use With or Without Opioid Use and All-Cause Mortality in the United States, 1999-2015
Source: JAMA Netw Open. 2020 Dec 9;3(12):e2028557. doi: 10.1001/jamanetworkopen.2020.28557 (PMC7726637; doi:10.1001/jamanetworkopen.2020.28557)
Supplement: Supplement. — eMethods. Overview of Propensity Score Derivation eTable 1. Classification Scheme of Benzodiazepines and Opioids eTable 2. Propensity Score Diagnostics for All Participants, as Well as Stratified by Age and Follow-up Time eTable 3. Follow-up Time and Event Rates Among Study Participants eTable 4. Sensitivity Analyses Depicting Risk of All-Cause Mortality Associated With Benzodiazepine/Opioid Co-Treatment and Benzodiazepine Monotherapy, With Removal of Non–Central Nervous System Medications eTable 5. Sensitivity Analyses Depicting Risk of All-Cause Mortality Associated With Benzodiazepine/Opioid Co-Treatment and Benzodiazepine Monotherapy, With Inclusion of Participants Who Died Within 1 Year [file jamanetwopen-e2028557-s001.pdf]

Supplementary Online Content

Xu KY, Hartz SM, Borodovsky JT, Bierut LJ, Grucza RA. Association between benzodiazepine use with or without opioid use and all-cause mortality in the United States, 1999-2015. *JAMA Netw Open.* 2020;3(12):e2028557. doi:10.1001/jamanetworkopen.2020.28557

eMethods. Overview of Propensity Score Derivation

eTable 1. Classification Scheme of Benzodiazepines and Opioids

eTable 2. Propensity Score Diagnostics for All Participants, as Well as Stratified by Age and Follow-up Time

eTable 3. Follow-up Time and Event Rates Among Study Participants

eTable 4. Sensitivity Analyses Depicting Risk of All-Cause Mortality Associated With Benzodiazepine/Opioid Co-Treatment and Benzodiazepine Monotherapy, With Removal of Non–Central Nervous System Medications

eTable 5. Sensitivity Analyses Depicting Risk of All-Cause Mortality Associated With Benzodiazepine/Opioid Co-Treatment and Benzodiazepine Monotherapy, With Inclusion of Participants Who Died Within 1 Year

This supplementary material has been provided by the authors to give readers additional information about their work.

© 2020 Xu KY et al. *JAMA Network Open.*

eMethods. Overview of Propensity Score Derivation

To develop propensity score weights for BZD exposure categories, we utilized data on baseline patient characteristics at time of study enrollment in order to predict probability of BZD prescription status. These included sociodemographic characteristics, comorbidities and other health-related variables and other prescriptions. A complete listing of covariates are in eTable 2.

Sociodemographic characteristics included age in years, sex, race (Hispanic, non-Hispanic White, non-Hispanic Black, and non-Hispanic other race), income-to-poverty ratio (with a ratio of "1" denoting a family income at 100% of the federal poverty level), highest education level attained (less than high school; high school graduate/general education degree or equivalent; some college or associate's degree; college graduate or above), partnered status. Health-related variables included, body mass index in kg/m^2 , current cigarette smoking, self-reported medical comorbidities (hypertension, hyperlipidemia, stroke, myocardial infarction, diabetes mellitus, congestive heart failure, asthma, bronchitis, congestive obstructive pulmonary disease, liver disease, renal disease, arthritis, cancer), general health and functional status, and healthcare utilization data (hospitalization in the <1 year, requirement of special health equipment, disability, regular emergency room visits, overnight hospitalizations >2 in the last year) in order to encompass both overt and often unmeasured risk factors for mortality.

Beyond the aforementioned data on psychotropic medications, mental health variables included visit to mental health professional in <1 year. Regular alcohol use was also included, defined as consuming alcohol on average ≥1 times per week in the past year, (i.e., at least 52 times in the past year).

Lexicon Plus® was used by NHANES to derive classifications for >1,000 unique prescription medications, spanning both central nervous system (CNS)- and non-CNS related classes. CNS-acting medications included antidepressants (selective serotonin reuptake inhibitors, serotonin and norepinephrine reuptake inhibitors, tricyclic antidepressants, monoamine inhibitors, and others), antipsychotics (first-generation, second-generation, and all other subtypes), barbiturates, anticonvulsants (which included common mood stabilizers), antihistamines, muscle relaxants, stimulants, Parkinson disease medications, antiemetics, non-opioid analgesics (including nonsteroidal anti-inflammatory medications), and other anticholinergics. Non CNS-acting agents included respiratory medications, genitourinary agents, endocrine agents and hormones (including steroids), cardiovascular medications, metabolic medications (including diabetes and cholesterol-lowering medications), and anti-microbial agents. To quantify potential polypharmacy burden, we tabulated the total number of CNS and non-CNS medications for each participant.

Using multivariable logistic regression models containing covariates, we calculated separate propensity scores in comparing the active comparator control group with exposure groups (1) and (2) individually. Since many comorbidities vary strongly by age, we included a number of age by comorbidity

© 2020 Xu KY et al. *JAMA Network Open.*

interactions in our logistic regression models. Where PS represents the propensity score for membership in that group, each treatment was weighted by (1-PS)/PS, and the control group is assigned a weight of 1. This results in an estimate of the treatment effects (i.e., effects of BZD/Opioid exposure) in a population resembling those who were treated with SSRIs. Since each treatment group is weighted to a single reference (in our case, the control group) population, effect-size estimates can be compared across treatment groups. We used standardized-mortality ratio weighting, allowing comparison of effect sizes across multiple treatment groups. Standardized differences were used to compare balance in baseline covariates with and without propensity score weighting, with results illustrated in eTable 2, with a threshold of < 0.1 to denote negligible difference in covariate balance between groups.[a,b]

Covariate data were missing in fewer than 5% of cases for all covariates with the exception of self-reported hyperlipidemia (38.8% did not recall previous cholesterol lab measurements), self-reported emergency room care (15.1% did not provide data on the type of place they most often frequent for healthcare), and income to poverty ratio (7.7% did not provide information on income). Multiple imputation (with 100 iterations) via the Markov Chain Monte Carlo method was utilized in the calculation of propensity scores for the aforementioned three variables in addition to partnered status, body mass index, and diabetes history.

[a]. Austin PC. Using the Standardized Difference to Compare the Prevalence of a Binary Variable Between Two Groups in Observational Research. *Communications in Statistics - Simulation and Computation* 2009;38(6):1228-34.

[b] Patorno E, Glynn RJ, Levin R, et al. Benzodiazepines and risk of all cause mortality in adults: cohort study. *BMJ* 2017;358:j2941. doi: 10.1136/bmj.j2941

© 2020 Xu KY et al. *JAMA Network Open.*

eTable 1.[a] Classification Scheme of Benzodiazepines (left) and Opioids (right)

BENZODIAZEPINES			OPIOIDS	
HYPNOTIC AGENTS				
d00238	FLURAZEPAM		a10129	CODEINE
d00917	QUAZEPAM		d00012	CODEINE
a56545	NITRAZEPAM		d00017	MEPERIDINE
d00384	TEMAZEPAM		d00050	METHADONE
			d00233	FENTANYL
ANXIOLYTIC			d00255	HYDROMORPHONE
d00189	CHLORDIAZEPOXIDE		d00308	MORPHINE
d03492	CHLORDIAZEPOXIDE; CLIDINIUM		d00329	OXYCODONE
h00012	CHLORDIAZEPOXIDE; ESTERIFIED ESTROGENS		d00334	PENTAZOCINE
d05416	CHLORDIAZEPOXIDE; METHSCOPOLAMINE		d00360	PROPOXYPHENE
d03462	AMITRIPTYLINE; CHLORDIAZEPOXIDE		d00824	OPIUM
d00198	CLORAZEPATE		d00833	OXYMORPHONE
d00148	DIAZEPAM		d00840	BUPRENORPHINE
a54760	PRAZEPAM		d03075	HYDROCODONE
d04557	CLOBAZAM		d03340	HOMATROPINE; HYDROCODONE
d00197	CLONAZEPAM		d03346	ACETAMINOPHEN; CAFFEINE; CHLORPHENIRAMINE; HYDROCODONE; PHENYLEPHRINE
d00149	LORAZEPAM		d03352	HYDROCODONE; PSEUDOEPHEDRINE
d00168	ALPRAZOLAM		d03353	HYDROCODONE; PHENYLPROPANOLAMIN E
d00040	OXAZEPAM		d03356	CHLORPHENIRAMINE; HYDROCODONE
h00001	BROMAZEPAM		d03357	CODEINE; PROMETHAZINE
d00397	TRIAZOLAM		d03361	CHLORPHENIRAMINE; HYDROCODONE; PHENYLEPHRINE
d00915	ESTAZOLAM		d03362	CHLORPHENIRAMINE; CODEINE; PSEUDOEPHEDRINE
			d03363	CODEINE; PSEUDOEPHEDRINE; TRIPROLIDINE
Z DRUGS			d03364	CODEINE; PHENYLEPHRINE; PROMETHAZINE

© 2020 Xu KY et al. *JAMA Network Open.*

d00910	ZOLPIDEM	d03366	HYDROCODONE; PHENYLEPHRINE; PYRILAMINE
d04452	ZALEPLON	d03367	BROMPHENIRAMINE; CODEINE; PHENYLPROPANOLAMINE
d07994	ESZOPICLONE	d03375	HYDROCODONE; PHENIRAMINE; PHENYLEPHRINE; PHENYLPROPANOLAMINE; PYRILAMINE
		d03393	CODEINE; GUAIFENESIN
		d03394	CODEINE; IODINATED GLYCEROL
		d03396	GUAIFENESIN; HYDROCODONE
		d03398	CODEINE; GUAIFENESIN; PSEUDOEPHEDRINE
		d03399	CHLORPHENIRAMINE; CODEINE; PHENYLEPHRINE; POTASSIUM IODIDE
		d03403	GUAIFENESIN; HYDROCODONE; PHENYLEPHRINE
		d03404	GUAIFENESIN; HYDROCODONE; PSEUDOEPHEDRINE
		d03407	CODEINE; GUAIFENESIN; PHENYLPROPANOLAMINE
		d03416	CHLORPHENIRAMINE; HYDROCODONE; PSEUDOEPHEDRINE
		d03423	ACETAMINOPHEN; CODEINE
		d03424	ASPIRIN; CODEINE
		d03425	ACETAMINOPHEN; BUTALBITAL; CAFFEINE; CODEINE
		d03426	ASPIRIN; BUTALBITAL; CAFFEINE; CODEINE
		d03428	ACETAMINOPHEN; HYDROCODONE
		d03429	ASPIRIN; HYDROCODONE
		d03430	ASPIRIN; CAFFEINE; DIHYDROCODEINE
		d03431	ACETAMINOPHEN; OXYCODONE
		d03432	ASPIRIN; OXYCODONE
		d03433	MEPERIDINE; PROMETHAZINE

© 2020 Xu KY et al. *JAMA Network Open.*

		d03434	ACETAMINOPHEN; PROPOXYPHENE
		d03435	ASPIRIN; CAFFEINE; PROPOXYPHENE
		d03436	BELLADONNA; OPIUM
		d03470	ASPIRIN; CARISOPRODOL; CODEINE
		d03576	BROMODIPHENHYDRAMINE; CODEINE
		d03630	ANHYDROUS CALCIUM IODIDE; CODEINE
		d03676	NALOXONE; PENTAZOCINE
		d03682	ACETAMINOPHEN; PENTAZOCINE
		d03826	TRAMADOL
		d03915	HYDROCODONE; POTASSIUM GUAIACOLSULFONATE
		d04152	HYDROCODONE; PHENYLEPHRINE
		d04225	HYDROCODONE; IBUPROFEN
		d04269	ACETAMINOPHEN; CAFFEINE; DIHYDROCODEINE
		d04752	CARBINOXAMINE; HYDROCODONE; PSEUDOEPHEDRINE
		d04766	ACETAMINOPHEN; TRAMADOL
		d04819	BUPRENORPHINE; NALOXONE
		d04870	DIHYDROCODEINE; GUAIFENESIN; PSEUDOEPHEDRINE
		d04880	BROMPHENIRAMINE; HYDROCODONE; PSEUDOEPHEDRINE
		d04904	CHLORPHENIRAMINE; DIHYDROCODEINE; PHENYLEPHRINE
		d04925	DIPHENHYDRAMINE; HYDROCODONE; PHENYLEPHRINE
		d05426	BROMPHENIRAMINE; HYDROCODONE; PHENYLEPHRINE
		d06058	DEXBROMPHENIRAMINE; HYDROCODONE; PHENYLEPHRINE

© 2020 Xu KY et al. *JAMA Network Open.*

		d06669	HYDROCODONE; PSEUDOEPHEDRINE; TRIPROLIDINE
		d07453	TAPENTADOL
		h00008	BELLADONNA; KAOLIN; PAREGORIC; PECTIN
		h00018	KAOLIN; PAREGORIC; PECTIN

a Medication names were matched to the Multum Lexicon prescription drug database, Lexicon Plus® by Cerner Multum, Inc. This was used to classify medications by therapeutic drug categories. Each generic medication is associated with a unique code from the Multum Lexicon prescription database. Generic drug codes from the database always begin with a "d". For medication names reported by participants that were not found in the database, unique codes beginning with "a" or "h" were subsequently assigned, whereas unspecified products with known therapeutic action were assigned a "c." There are 1,478 unique generic medication codes total in NHANES, with 1,352 beginning with "d", 33 with "a," 39 with "h," and 55 with "c." This data was stored in an event-level database such that each participant may have multiple prescriptions. For additional information on Multum Lexicon, please refer to the following websites: https://www.cerner.com/solutions/drug-database and https://wwwn.cdc.gov/Nchs/Nhanes/1999-2000/RXQ_DRUG.htm.

© 2020 Xu KY et al. *JAMA Network Open.*

eTable 2. Propensity Score Diagnostics for All Participants, as Well as Stratified by Age and Follow-up Time

eTable 2A. Propensity score diagnostics for all participants

Propensity score diagnostics for all participants for all variables included in propensity score

	BZDs [a] vs Neither (active comparator, SSRIs)		BZDs+Opioids vs Neither (active comparator, SSRIs)	
ALL AGES	STDDIFF		STDDIFF	
	after PS	before PS	after PS	before PS
Age 60-70	0.02	-0.01	0.03	0.07
Male Sex	0.01	0.1	0.05	0.1
College Education	-0.02	-0.1	0.02	-0.32
Poverty to Income 1-2	0.01	0.08	0.04	0.12
Poverty to Income >2	-0.02	-0.11	0	-0.41
White	-0.01	-0.04	-0.03	-0.03
Partnered	-0.02	-0.06	0.01	-0.08
Smoking	0.02	0.03	-0.06	0.31
Hypertension	-0.01	0.03	0.11	0.19
Hyperlipidemia	-0.02	-0.01	-0.04	-0.03
Stroke	0.02	-0.03	-0.01	0.11
Myocardial Infarction	0.02	0.04	-0.02	0.12
Diabetes Mellitus	-0.03	-0.03	0	0.07
Congestive Heart Failure	0	0.04	0.02	0.12
Pulmonary Disease	-0.02	-0.02	-0.02	0.23
Liver Disease	0.02	0.03	0.07	0.13
Arthritis	0	0.08	0.11	0.61
Renal Disease	-0.01	0.06	0	0.15
Cancer	0.03	0.15	0.1	0.23
Regular drinking	0.01	-0.05	0.09	-0.21
Antimicrobial Medications	-0.01	0.05	0.05	0.22
Hormonal Agents	-0.05	0.03	-0.1	0.04
Anticonvulsants	0.03	0.09	-0.05	0.37
Any Analgesics	0	0.06	0.1	0.25
Muscle Relaxants	0.04	0.11	0.07	0.58
Gastrointestinal Agents	0	0.09	0.07	0.34
Cardiac, Metabolic Medications	-0.02	0.12	0.12	0.23
Respiratory Medications /Antihistamines	-0.02	0.02	0.13	0.31
>2 CNS [b] Medications	0.04	0.19	0.09	0.59

© 2020 Xu KY et al. *JAMA Network Open.*

<5 Non CNS Medications	-0.02	0.03	0.1	0.02
Antidepressants (SNRI, MAOI, TCA) [c]	0	0.31	0	0.45
Other Antidepressants	-0.01	0.06	-0.01	0.09
Antipsychotics	-0.01	0.04	0.01	0.02
Any Hospitalization in <1 year	-0.02	0.13	0.06	0.34
Any Psychiatric Visit in <1 year	-0.02	0	0.07	-0.02
Good Current Health	-0.07	-0.13	-0.02	-0.59
Require Special Health Equipment	0.04	0.01	0.04	0.46
Disabled	0.02	0.14	-0.06	0.74
Regular ER Care [d]	0	0.02	-0.01	0.06
Worsening Health	0	0.15	-0.02	0.38
Overnight Hospitalization >2 in <1 year	-0.01	0.06	0	0.21

Propensity score diagnostics for all participants, after removing >650 non-CNS medications, including all cardiac, metabolic, hormonal, and antimicrobial agents

	BZDs vs Neither (active comparator, SSRIs)		BZDs+Opioids vs Neither (active comparator, SSRIs)	
ALL AGES	STDDIFF		STDDIFF	
	after PS	before PS	after PS	before PS
Age 60-70	0.01	-0.01	0.04	0.09
Male Sex	0.01	0.1	0.05	0.1
College Education	-0.02	-0.1	0.01	-0.32
Poverty to Income <2	0.01	0.08	0.06	0.13
Poverty to Income >2	-0.02	-0.12	-0.02	-0.41
White	-0.01	-0.04	-0.05	-0.03
Partnered	-0.02	-0.06	-0.02	-0.09
Smoking	0.02	0.03	-0.06	0.32
Hypertension	-0.01	0.02	0.1	0.2
Hyperlipidemia	-0.02	-0.01	-0.04	-0.03
Stroke	0.02	-0.03	0	0.12
Myocardial Infarction	0.02	0.04	-0.01	0.12
Diabetes Mellitus	-0.03	-0.03	-0.01	0.06
Congestive Heart Failure	0	0.03	0.02	0.13
Pulmonary Disease	-0.03	-0.02	-0.03	0.24
Liver Disease	0.02	0.03	0.07	0.15
Arthritis	0	0.08	0.13	0.61
Renal Disease	0	0.06	0.01	0.16
Cancer	0.02	0.15	0.09	0.23
Regular drinking	0.01	-0.05	0.08	-0.22

© 2020 Xu KY et al. *JAMA Network Open.*

Anticonvulsants	0.03	0.09	-0.04	0.38
Any Analgesics	-0.01	0.06	0.1	0.26
Muscle Relaxants	0.04	0.11	0.08	0.6
Gastrointestinal Agents	0	0.09	0.08	0.34
Respiratory Medications /Antihistamines	-0.02	0.02	0.11	0.31
>2 CNS Medications	0.04	0.19	0.08	0.61
<5 Non CNS Medications	-0.02	0.03	0.09	0.04
Antidepressants (SNRI, MAOI, TCA)	0	0.31	0.01	0.45
Other Antidepressants	-0.01	0.06	-0.03	0.11
Antipsychotics	-0.01	0.03	-0.01	0.02
Any Hospitalization in <1 year	-0.02	0.13	0.08	0.34
Any Psychiatric Visit in <1 year	-0.02	0	0.05	-0.02
Good Current Health	-0.08	-0.14	-0.01	-0.6
Require Special Health Equipment	0.04	0.01	0.03	0.46
Disabled	0.01	0.14	-0.04	0.75
Regular ER Care	0	0.02	-0.02	0.06
Worsening Health	0	0.16	0	0.39
Overnight Hospitalization >2 in <1 year	-0.01	0.06	0	0.22

eTable 2B. Propensity score diagnostics, ages 20-65

Propensity score diagnostics, ages 20-65, for all variables included in propensity score

	BZDs vs Neither (active comparator, SSRIs)		BZDs+Opioids vs Neither (active comparator, SSRIs)	
Age 20-65	STDDIFF		STDDIFF	
	after PS	before PS	after PS	before PS
Age 60-70	0.05	0.01	0.09	0.01
Male Sex	0	0.13	0.06	0.16
College Education	-0.04	-0.09	0.01	-0.4
Poverty to Income <2	0.02	0.09	0.07	0.19
Poverty to Income >2	-0.05	-0.13	0	-0.56
White	-0.02	-0.08	-0.1	-0.06
Partnered	-0.01	-0.07	0.04	-0.14
Smoking	0.03	0.1	-0.04	0.36
Hypertension	0.03	0.01	0.12	0.28
Hyperlipidemia	-0.02	-0.02	-0.13	-0.01
Stroke	0.03	0.01	0.02	0.19

© 2020 Xu KY et al. *JAMA Network Open.*

Myocardial Infarction	0	0.04	0	0.18
Diabetes Mellitus	0	-0.07	0.01	0.07
Congestive Heart Failure	-0.01	0.03	-0.06	0.13
Pulmonary Disease	-0.02	0	-0.07	0.26
Liver Disease	0.02	0.09	0.14	0.2
Arthritis	0.01	0.1	0.11	0.71
Renal Disease	0	0.05	-0.01	0.16
Cancer	0.02	0.09	0.07	0.29
Regular drinking	0.01	-0.02	0	-0.23
Antimicrobial Medications	-0.03	0.08	0.1	0.26
Hormonal Agents	-0.02	-0.02	-0.11	-0.03
Anticonvulsants	0.02	0.17	-0.11	0.41
Any Analgesics	0.03	0.07	0.14	0.35
Muscle Relaxants	0.02	0.19	0.05	0.71
Gastrointestinal Agents	-0.02	0.1	0.09	0.38
Cardiac, Metabolic Medications	0.01	0.09	0.02	0.28
Respiratory Medications/ Antihistamines	-0.02	0.03	0.06	0.34
>2 CNS Medications	0.04	0.27	0.1	0.71
<5 Non CNS Medications	0.01	-0.03	0.09	0.05
Antidepressants (SNRI, MAOI, TCA)	0	0.35	0.03	0.49
Other Antidepressants	-0.01	0.09	0	0.09
Antipsychotics	-0.01	0.11	0.03	0.02
Any Hospitalization in <1 year	0	0.1	0.05	0.42
Any Psychiatric Visit in <1 year	-0.02	0.08	0.05	-0.02
Good Current Health	-0.04	-0.19	-0.06	-0.65
Require Special Health Equipment	0.02	0.08	0.01	0.59
Disabled	0.03	0.23	0.01	0.87
Regular ER Care	0.01	0.07	0.02	0.07
Worsening Health	-0.01	0.2	-0.06	0.42
Overnight Hospitalization >2 in <1 year	0	0.06	-0.07	0.29

Propensity score diagnostics, ages 20-65, after removing >650 non-CNS medications, including all cardiac, metabolic, hormonal, and antimicrobial agents,

	BZDs vs Neither (active comparator, SSRIs)	BZDs+Opioids vs Neither (active comparator, SSRIs)
Age 20-65	STDDIFF	STDDIFF

© 2020 Xu KY et al. *JAMA Network Open.*

	after PS	before PS	after PS	before PS
Age 60-70	0.03	0	0.11	0.02
Male Sex	0.01	0.13	0.06	0.16
College Education	-0.04	-0.09	0.03	-0.4
Poverty to Income <2	0.02	0.08	0.07	0.18
Poverty to Income >2	-0.05	-0.13	0.01	-0.57
White	-0.02	-0.08	-0.1	-0.06
Partnered	-0.01	-0.06	0.04	-0.16
Smoking	0.03	0.11	-0.06	0.37
Hypertension	0.03	0.01	0.1	0.29
Hyperlipidemia	-0.01	-0.02	-0.11	0.01
Stroke	0.03	0.01	0.01	0.2
Myocardial Infarction	0.01	0.04	-0.02	0.17
Diabetes Mellitus	-0.01	-0.08	0.02	0.09
Congestive Heart Failure	-0.01	0.03	-0.04	0.14
Pulmonary Disease	-0.02	0	-0.08	0.26
Liver Disease	0.01	0.09	0.12	0.21
Arthritis	0.01	0.11	0.12	0.72
Renal Disease	0.01	0.05	-0.01	0.18
Cancer	0.02	0.1	0.05	0.3
Regular drinking	0.01	-0.02	0.02	-0.23
Anticonvulsants	0.02	0.18	-0.11	0.43
Any Analgesics	0.03	0.07	0.13	0.35
Muscle Relaxants	0.03	0.2	0.05	0.72
Gastrointestinal Agents	-0.02	0.1	0.08	0.39
Respiratory Medications /Antihistamines	-0.02	0.03	0.04	0.34
>2 CNS Medications	0.03	0.28	0.07	0.72
<5 Non CNS Medications	0.01	-0.04	0.11	0.06
Antidepressants (SNRI, MAOI, TCA)	0.01	0.37	0.02	0.5
Other Antidepressants	-0.02	0.09	0	0.1
Antipsychotics	-0.02	0.11	0.02	0.03
Any Hospitalization in <1 year	0	0.12	0.04	0.41
Any Psychiatric Visit in <1 year	-0.01	0.08	0.04	-0.01
Good Current Health	-0.05	-0.19	-0.05	-0.66
Require Special Health Equipment	0.02	0.08	0.01	0.59
Disabled	0.03	0.24	-0.01	0.87
Regular ER Care	0	0.07	0.01	0.07
Worsening Health	0	0.21	-0.07	0.42

© 2020 Xu KY et al. *JAMA Network Open.*

Overnight Hospitalization >2 in <1 year	0	0.06	-0.07	0.29

eTable 2C. Propensity score diagnostics, age 65 and over

Propensity score diagnostics, age 65 and over, for all variables included in propensity score

	BZDs vs Neither (active comparator, SSRIs)		BZDs+Opioids vs Neither (active comparator, SSRIs)	
Age 65 and over	STDDIFF		STDDIFF	
	after PS	before PS	after PS	before PS
Age 60-70	-0.04	-0.07	-0.02	0.24
Male Sex	-0.01	-0.01	-0.04	-0.06
College Education	0	-0.04	-0.11	-0.11
Poverty to Income <2	0.01	-0.02	-0.05	0.01
Poverty to Income >2	0.01	-0.03	0.05	-0.06
White	0.03	0	0.09	0.01
Partnered	0.02	0	-0.04	0.05
Smoking	0.01	0.01	-0.02	0.23
Hypertension	0	-0.08	0.2	0.01
Hyperlipidemia	-0.01	-0.06	0.1	-0.13
Stroke	0.02	-0.12	-0.16	-0.04
Myocardial Infarction	0.04	-0.06	-0.14	0.02
Diabetes Mellitus	0	-0.03	-0.03	0.03
Congestive Heart Failure	0.02	-0.04	0.03	0.1
Pulmonary Disease	-0.02	-0.02	0	0.2
Liver Disease	-0.02	-0.06	-0.1	-0.03
Arthritis	-0.03	-0.13	0	0.37
Renal Disease	-0.04	0.03	-0.12	0.13
Cancer	0.04	0.12	0.12	0.11
Regular drinking	0	-0.04	0.06	-0.16
Antimicrobial Medications	0.03	0	-0.05	0.15
Hormonal Agents	-0.03	0.08	-0.07	0.22
Anticonvulsants	-0.02	-0.01	-0.01	0.23
Any Analgesics	0.01	0.02	-0.05	0.02
Muscle Relaxants	0.04	-0.07	0.07	0.23
Gastrointestinal Agents	0	-0.04	-0.06	0.23
Cardiac, Metabolic Medications	-0.01	-0.11	0	0.01

© 2020 Xu KY et al. *JAMA Network Open.*

Respiratory Medications /Antihistamines	-0.02	-0.01	0.08	0.27
>2 CNS Medications	0	0.04	-0.04	0.29
<5 Non CNS Medications	0.01	0.06	-0.08	0.03
Antidepressants (SNRI, MAOI, TCA)	-0.01	0.2	-0.05	0.36
Other Antidepressants	0.02	0.08	0.04	0.15
Antipsychotics	0	-0.06	-0.09	0.01
Any Hospitalization in <1 year	-0.02	0.1	0.09	0.14
Any Psychiatric Visit in <1 year	0.03	0.02	0.03	-0.03
Good Current Health	-0.02	-0.01	0.07	-0.43
Require Special Health Equipment	0.05	-0.18	0.13	0.22
Disabled	0.01	-0.01	-0.08	0.46
Regular ER Care	0.02	-0.01	-0.11	0.02
Worsening Health	0	0.06	-0.12	0.3
Overnight Hospitalization >2 in <1 year	-0.04	0.02	-0.03	0.04

Propensity score diagnostics, age 65 and over, after removing >650 non-CNS medications, including all cardiac, metabolic, hormonal, and antimicrobial agents,

	BZDs vs Neither (active comparator, SSRIs)		BZDs+Opioids vs Neither (active comparator, SSRIs)	
Age 65 and over	STDDIFF		STDDIFF	
	after PS	before PS	after PS	before PS
Age 60-70	-0.03	-0.06	0	0.25
Male Sex	0	-0.01	0.03	-0.05
College Education	0.01	-0.04	-0.05	-0.12
Poverty to Income <2	-0.01	-0.02	0.03	0.03
Poverty to Income >2	0.01	-0.04	-0.05	-0.08
White	0.04	0.01	-0.02	0.02
Partnered	0.01	0	-0.15	0.04
Smoking	0.01	0.01	0.01	0.24
Hypertension	0	-0.08	0.05	0
Hyperlipidemia	0	-0.05	-0.03	-0.12
Stroke	-0.02	-0.15	-0.13	-0.04
Myocardial Infarction	0.03	-0.05	-0.05	0.05

© 2020 Xu KY et al. *JAMA Network Open.*

Diabetes Mellitus	-0.03	-0.03	0.03	0.02
Congestive Heart Failure	0.01	-0.04	0.04	0.13
Pulmonary Disease	-0.02	-0.02	0.07	0.2
Liver Disease	-0.01	-0.06	-0.11	-0.03
Arthritis	-0.04	-0.14	0.11	0.37
Renal Disease	-0.03	0.03	-0.1	0.13
Cancer	0.04	0.12	0.17	0.11
Regular drinking	-0.01	-0.05	0.08	-0.16
Anticonvulsants	-0.07	-0.06	0	0.24
Any Analgesics	0	0.02	-0.04	0.02
Muscle Relaxants	0	-0.13	0.06	0.26
Gastrointestinal Agents	0.03	-0.02	0.02	0.24
Respiratory Medications /Antihistamines	-0.01	-0.01	0.16	0.27
>2 CNS Medications	-0.02	0.01	-0.02	0.33
<5 Non CNS Medications	0.02	0.07	-0.03	0.03
Antidepressants (SNRI, MAOI, TCA)	0	0.21	-0.03	0.35
Other Antidepressants	0.01	0.08	0.04	0.15
Antipsychotics	-0.04	-0.1	-0.09	0.02
Any Hospitalization in <1 year	-0.01	0.11	0.12	0.15
Any Psychiatric Visit in <1 year	0.01	0	0	-0.05
Good Current Health	-0.03	-0.01	0	-0.44
Require Special Health Equipment	0	-0.22	-0.03	0.21
Disabled	0.01	-0.03	0	0.47
Regular ER Care	-0.01	-0.04	-0.11	0.01
Worsening Health	-0.02	0.04	-0.08	0.29
Overnight Hospitalization >2 in <1 year	-0.03	0.02	0.04	0.06

eTable 2D. Propensity score diagnostics, follow-up time under 50th percentile (6.58 years)

Propensity score diagnostics, follow-up time under 50th percentile (6.58 years), for all variables included in propensity score

	BZDs vs Neither (active comparator, SSRIs)		BZDs+Opioids vs Neither (active comparator, SSRIs)	
Follow-up under 50th percentile	STDDIFF		STDDIFF	
	after PS	before PS	after PS	before PS
Age 60-70	-0.01	-0.06	0.07	0.03
Male Sex	-0.02	0.05	0.07	0.06

© 2020 Xu KY et al. *JAMA Network Open.*

College Education	-0.02	-0.05	0.07	-0.32
Poverty to Income <2	0.02	0.05	0.04	0.14
Poverty to Income >2	-0.01	-0.07	-0.02	-0.4
White	0.01	0.01	0	0.03
Partnered	0	-0.06	-0.01	-0.06
Smoking	0	0.03	-0.03	0.34
Hypertension	-0.01	-0.1	0.18	0.1
Hyperlipidemia	-0.02	-0.03	-0.01	-0.02
Stroke	0.03	-0.02	-0.02	0.11
Myocardial Infarction	0.02	-0.01	-0.14	0.04
Diabetes Mellitus	-0.07	-0.13	-0.11	-0.01
Congestive Heart Failure	0	0.04	0.01	0.1
Pulmonary Disease	-0.02	0	0	0.23
Liver Disease	-0.01	0.04	0.07	0.18
Arthritis	0.01	-0.07	0.08	0.52
Renal Disease	-0.03	0	-0.04	0.06
Cancer	0.04	0.12	0.07	0.26
Regular drinking	0.01	-0.05	0.06	-0.19
Antimicrobial Medications	0	0.11	0.01	0.27
Hormonal Agents	-0.04	0.03	-0.15	0.11
Anticonvulsants	0.01	0.06	-0.08	0.42
Any Analgesics	-0.01	-0.02	0.06	0.24
Muscle Relaxants	0.04	0	0.03	0.52
Gastrointestinal Agents	-0.01	0.01	0.02	0.23
Cardiac, Metabolic Medications	-0.04	-0.03	0.04	0.09
Respiratory Medications /Antihistamines	-0.05	0.01	0.01	0.32
>2 CNS Medications	0.03	0.11	0.08	0.58
<5 Non CNS Medications	-0.01	0.01	0.12	0.02
Antidepressants (SNRI, MAOI, TCA)	-0.01	0.31	0	0.41
Other Antidepressants	-0.01	0.04	0.03	0.08
Antipsychotics	-0.04	0.03	0.06	-0.01
Any Hospitalization in <1 year	-0.02	0.1	0.03	0.27
Any Psychiatric Visit in <1 year	0	0.08	0.27	0.02
Good Current Health	-0.04	-0.08	0.02	-0.57
Require Special Health Equipment	0.03	-0.04	-0.05	0.36
Disabled	0	0.1	0	0.64

© 2020 Xu KY et al. *JAMA Network Open.*

Regular ER Care	-0.01	0.04	-0.04	0.07
Worsening Health	-0.03	0.1	-0.05	0.34
Overnight Hospitalization >2 in <1 year	-0.02	0.08	-0.12	0.21

Propensity score diagnostics, follow-up time under 50th percentile (6.58 years), after removing >650 non-CNS medications, including all cardiac, metabolic, hormonal, and antimicrobial agents,

	BZDs vs Neither (active comparator, SSRIs)		BZDs+Opioids vs Neither (active comparator, SSRIs)	
Follow-up under 50th percentile	STDDIFF		STDDIFF	
	after PS	before PS	after PS	before PS
Age 60-70	-0.02	-0.06	0.09	0.02
Male Sex	-0.02	0.05	0.04	0.08
College Education	-0.01	-0.04	0.06	-0.29
Poverty to Income <2	0.01	0.06	0.07	0.16
Poverty to Income >2	0	-0.07	-0.03	-0.39
White	0	0.01	-0.05	0.04
Partnered	0	-0.06	-0.07	-0.08
Smoking	0	0.04	-0.03	0.34
Hypertension	-0.01	-0.1	0.13	0.11
Hyperlipidemia	-0.02	-0.03	0.01	-0.02
Stroke	0.03	-0.01	-0.01	0.11
Myocardial Infarction	0	-0.02	-0.08	0.06
Diabetes Mellitus	-0.07	-0.13	-0.12	-0.01
Congestive Heart Failure	-0.01	0.04	-0.01	0.12
Pulmonary Disease	-0.03	0	0.01	0.24
Liver Disease	-0.01	0.03	0.1	0.2
Arthritis	0	-0.08	0.11	0.51
Renal Disease	-0.03	0	-0.03	0.06
Cancer	0.03	0.12	0.08	0.25
Regular drinking	0.01	-0.05	0.06	-0.19
Anticonvulsants	0.01	0.05	-0.06	0.43
Any Analgesics	-0.01	-0.02	0.07	0.24
Muscle Relaxants	0.03	-0.01	0.07	0.55
Gastrointestinal Agents	-0.01	0.01	0.03	0.25
Respiratory Medications /Antihistamines	-0.05	0.01	0.02	0.33
>2 CNS Medications	0.02	0.1	0.07	0.61
<5 Non CNS Medications	-0.01	0.02	0.1	0.04

© 2020 Xu KY et al. *JAMA Network Open.*

Antidepressants (SNRI, MAOI, TCA)	-0.02	0.29	-0.01	0.42
Other Antidepressants	-0.02	0.04	0.01	0.11
Antipsychotics	-0.03	0.03	0.06	-0.02
Any Hospitalization in <1 year	-0.01	0.1	0.13	0.29
Any Psychiatric Visit in <1 year	0	0.08	0.21	0.04
Good Current Health	-0.04	-0.08	0.02	-0.57
Require Special Health Equipment	0.02	-0.05	-0.04	0.37
Disabled	0	0.09	0.02	0.64
Regular ER Care	-0.01	0.04	-0.06	0.06
Worsening Health	-0.03	0.09	0	0.36
Overnight Hospitalization >2 in <1 year	-0.01	0.09	-0.08	0.22

eTable 2E**.** Propensity score diagnostics, follow-up time above 50th percentile (6.58 years)

Propensity score diagnostics, follow-up time above 50th percentile (6.58 years), for all variables included in propensity score

	BZDs vs Neither (active comparator, SSRIs)		BZDs+Opioids vs Neither (active comparator, SSRIs)	
Follow-up time above 50th percentile	STDDIFF		STDDIFF	
	after PS	before PS	after PS	before PS
Age 60-70	0.04	0.05	-0.01	0.11
Male Sex	0.01	0.15	-0.01	0.05
College Education	0	-0.16	0.05	-0.2
Poverty to Income <2	0.01	0.09	0.07	0.08
Poverty to Income >2	-0.02	-0.14	0.06	-0.33
White	-0.02	-0.09	0.04	-0.05
Partnered	-0.01	-0.04	-0.07	-0.08
Smoking	0.03	0.03	-0.1	0.24
Hypertension	-0.04	0.15	0.11	0.22
Hyperlipidemia	-0.01	0.03	0	0.02
Stroke	0	-0.03	0.02	0.11
Myocardial Infarction	-0.01	0.1	0.05	0.19
Diabetes Mellitus	0	0.08	0.04	0.11
Congestive Heart Failure	-0.04	0.03	0	0.09
Pulmonary Disease	-0.04	-0.03	-0.17	0.17
Liver Disease	0.03	0.01	0.06	0.04
Arthritis	-0.02	0.21	0.19	0.57

© 2020 Xu KY et al. *JAMA Network Open.*

Renal Disease	0.01	0.15	0.02	0.22
Cancer	0.02	0.18	0.11	0.15
Regular drinking	0.02	-0.04	0.19	-0.16
Antimicrobial Medications	0.02	0	0.14	0.12
Hormonal Agents	-0.03	0.03	-0.03	0.03
Anticonvulsants	0.04	0.11	-0.06	0.19
Any Analgesics	-0.01	0.12	0.17	0.26
Muscle Relaxants	0.03	0.22	0.02	0.59
Gastrointestinal Agents	-0.01	0.17	0.16	0.38
Cardiac, Metabolic Medications	-0.01	0.25	0.05	0.29
Respiratory Medications /Antihistamines	0	0.02	0.05	0.2
>2 CNS Medications	0.04	0.25	0.06	0.52
<5 Non CNS Medications	-0.04	0.05	0.03	0.02
Antidepressants (SNRI, MAOI, TCA)	0.01	0.28	-0.01	0.42
Other Antidepressants	-0.02	0.07	0.03	0.1
Antipsychotics	0.03	0.03	-0.17	0.06
Any Hospitalization in <1 year	-0.02	0.15	-0.03	0.39
Any Psychiatric Visit in <1 year	-0.05	-0.09	-0.09	-0.03
Good Current Health	-0.09	-0.17	0.07	-0.49
Require Special Health Equipment	0.06	0.06	-0.04	0.51
Disabled	0.02	0.15	-0.16	0.71
Regular ER Care	0.01	0	-0.04	0.02
Worsening Health	0.02	0.2	-0.06	0.35
Overnight Hospitalization >2 in <1 year	0.03	0.02	0.05	0.21

Propensity score diagnostics, follow-up time above 50th percentile (6.58 years), after removing >650 non-CNS medications, including all cardiac, metabolic, hormonal, and antimicrobial agents,

	BZDs vs Neither (active comparator, SSRIs)		BZDs+Opioids vs Neither (active comparator, SSRIs)	
Follow-up time above 50th percentile	STDDIFF		STDDIFF	
	after PS	before PS	after PS	before PS
Age 60-70	0.03	0.05	-0.02	0.14
Male Sex	0.02	0.16	-0.01	0.06
College Education	0	-0.17	0.05	-0.25
Poverty to Income <2	0.01	0.09	0.06	0.08

© 2020 Xu KY et al. *JAMA Network Open.*

Poverty to Income >2	-0.01	-0.14	0.05	-0.36
White	-0.02	-0.09	0.02	-0.06
Partnered	-0.03	-0.04	-0.09	-0.09
Smoking	0.03	0.03	-0.11	0.25
Hypertension	-0.03	0.16	0.05	0.24
Hyperlipidemia	-0.02	0.03	-0.06	0.02
Stroke	0.01	-0.03	-0.05	0.09
Myocardial Infarction	0	0.1	0	0.17
Diabetes Mellitus	0	0.08	0.04	0.09
Congestive Heart Failure	-0.03	0.04	-0.01	0.09
Pulmonary Disease	-0.05	-0.03	-0.14	0.19
Liver Disease	0.05	0.02	0.02	0.04
Arthritis	-0.02	0.22	0.15	0.62
Renal Disease	0.01	0.15	0.02	0.26
Cancer	0.01	0.18	0.11	0.16
Regular drinking	0.02	-0.04	0.18	-0.17
Anticonvulsants	0.04	0.11	-0.07	0.19
Any Analgesics	-0.01	0.13	0.17	0.26
Muscle Relaxants	0.03	0.22	0.02	0.62
Gastrointestinal Agents	-0.01	0.18	0.12	0.41
Respiratory Medications /Antihistamines	0	0.02	0.07	0.21
>2 CNS Medications	0.04	0.25	0.05	0.54
<5 Non CNS Medications	-0.04	0.05	0.06	0.04
Antidepressants (SNRI, MAOI, TCA)	0.01	0.28	0	0.44
Other Antidepressants	-0.01	0.07	-0.01	0.09
Antipsychotics	0.03	0.03	-0.19	0.04
Any Hospitalization in <1 year	-0.02	0.15	-0.04	0.38
Any Psychiatric Visit in <1 year	-0.04	-0.1	-0.15	-0.05
Good Current Health	-0.1	-0.17	0.07	-0.53
Require Special Health Equipment	0.06	0.06	-0.02	0.51
Disabled	0.02	0.15	-0.15	0.76
Regular ER Care	0	0	-0.04	0.02
Worsening Health	0.02	0.2	-0.04	0.38
Overnight Hospitalization >2 in <1 year	0.02	0.02	0.04	0.19

© 2020 Xu KY et al. *JAMA Network Open.*

[a] Benzodiazepines (BZDs)

[b] Central nervous system (CNS)

[c] Serotonin norepinephrine reuptake inhibitor(SNRI), monoamine inhibitor (MAOI), tricyclic antidepressant (TCA)

[d] Emergency room (ER)

© 2020 Xu KY et al. *JAMA Network Open.*

eTable 3. Follow-up Time and Event Rates Among Study Participants

	Median follow-up (years)	Mean follow-up (years)	Events	Person years	Events per 1,000 Person years
**All Participants, excluding deaths in <1year** N=5,212	6.67	7.21	892	37,578.5	23.74
**Benzodiazepines +/- opioids**, N=1,724	6.33	6.93	337	11,947.3	28.21
BZDs + opioids, N=468	6.25	6.54	101	3,060.7	33.00
BZDs only, N=1,256	6.42	7.08	236	8,892.5	26.54
**No Benzodiazepines,** N=3,488	6.75	7.36	555	25,671.7	21.62
Opioids only, N=1,955	6.75	7.36	328	14,388.8	22.80
Neither (active comparator, SSRIs), N=1,533	6.75	7.35	227	11,267.6	20.15
**Stratified by Age**					
20-65 years old, N=3,575	7.25	7.68	284	27,456	10.34
> 65 years old, N=1,637	5.67	6.20	608	10,149.4	59.91

© 2020 Xu KY et al. *JAMA Network Open.*

eTable 4. Sensitivity Analyses Depicting Risk of All-Cause Mortality Associated With Benzodiazepine (BZD)/Opioid Co-Treatment and BZD Monotherapy, With Removal of Non–Central Nervous System (CNS) Medications

		**Unweighted**	**Weighted**	**Weighted (non-CNS medications removed from propensity score)**
**BZDs Only Versus Neither (active comparator, selective serotonin reuptake inhibitors [SSRIs])**				
All Participants		1.36 (1.13-1.64), p=0.001	1.60 (1.33-1.92), p<.0001	1.58 (1.29-1.94, p<.0001
Age	20-65 years old	1.52 (1.06-2.18), p=0.02	1.81 (1.29-2.54), p=0.0006	1.80 (1.26-2.58), p=0.001
	≥ 65 years old	0.86 (0.68-1.07), p=0.17	0.84 (0.67-1.05), p=0.12	0.87 (0.69-1.08), p=0.20
Follow-up Time	< 50th percentile (6.58 years)	1.03 (0.82-1.31), p=0.79	1.17 (0.92-1.50), p=0.21	1.17 (0.92-1.48), p=0.21
	≥ 50th percentile (6.58 years)	1.81 (1.31-2.50), p=0.0003	2.17 (1.59-2.98), p<.0001	2.17 (1.55-3.03), p<.0001
**BZDs + Opioids Versus Neither (active comparator, SSRIs)**				
All Participants		1.71 (1.34-2.19), p<.0001	2.04 (1.65-2.52), p<.0001	1.93 (1.57-2.36), p<.0001
Age	20-65 years old	2.66 (1.77-4.00), p<.0001	3.27 (2.40-4.47), p<.0001	3.38 (2.51-4.55), p<.0001
	≥ 65 years old	1.10 (0.80-1.51), p=0.55	1.21 (0.86-1.70), p=0.28	1.13 (0.87-1.48), p=0.35
Follow-up Time	< 50th percentile (6.58 years)	1.21 (0.90-1.63), p=0.20	1.35 (1.04-1.76), p=0.02	1.37 (1.06-1.77), p=0.02
	≥ 50th percentile (6.58 years)	1.73 (1.09-2.75), p=0.02	1.93 (1.29-2.88), p=0.002	2.07 (1.48-2.89), p<.0001

© 2020 Xu KY et al. *JAMA Network Open.*

eTable 5. Sensitivity Analyses Depicting Risk of All-Cause Mortality Associated With Benzodiazepine (BZD)/Opioid Co-Treatment and BZD Monotherapy, With Inclusion of Participants Who Died Within 1 Year

		**Weighted (excluding participants who died within 1 year)**	**Weighted (including participants who died within 1 year)**
**BZDs Only Versus Neither (selective serotonin reuptake inhibitors [SSRIs], active comparator)**			
All Participants		1.60 (1.33-1.92), p<.0001	1.60 (1.33-1.93), p<.0001
Age	20-65 years old	1.81 (1.29-2.54), p=0.0006	1.81 (1.31-2.52), p=0.0004
	≥ 65 years old	0.84 (0.67-1.05), p=0.12	0.87 (0.71-1.07), p=0.20
Follow-up Time	< 50th percentile (6.58 years)	1.17 (0.92-1.50), p=0.21	1.23 (0.99-1.54), p=0.06
	≥ 50th percentile (6.58 years)	2.17 (1.59-2.98), p<.0001	2.18 (1.55-4.07), p<.0001
**BZDs + Opioids Versus Neither (SSRI, active comparator)**			
All Participants		2.04 (1.65-2.52), p<.0001	1.63 (1.37-1.95), p<.0001
Age	20-65 years old	3.27 (2.40-4.47), p<.0001	2.92 (2.19-3.90), p<.0001
	≥ 65 years old	1.21 (0.86-1.70), p=0.28	1.13 (0.84-1.53), p=0.42
Follow-up Time	< 50th percentile (6.58 years)	1.35 (1.04-1.76), p=0.02	1.22 (0.78-1.53), p=0.09
	≥ 50th percentile (6.58 years)	1.93 (1.29-2.88), p=0.002	1.65 (1.07-2.55), p=0.02

© 2020 Xu KY et al. *JAMA Network Open.*
